# Vocabulary Knowledge Predicts Lexical Processing: Evidence from a Group of Participants with Diverse Educational Backgrounds

**DOI:** 10.3389/fpsyg.2017.01164

**Published:** 2017-07-13

**Authors:** Nina Mainz, Zeshu Shao, Marc Brysbaert, Antje S. Meyer

**Affiliations:** ^1^Psychology of Language Department, Max Planck Institute for Psycholinguistics Nijmegen, Netherlands; ^2^Department of Experimental Psychology, Ghent University Ghent, Belgium; ^3^Donders Institute for Brain, Cognition and Behaviour, Radboud University Nijmegen, Netherlands

**Keywords:** vocabulary, word knowledge, individual differences, lexical decision, vocabulary tests

## Abstract

Vocabulary knowledge is central to a speaker's command of their language. In previous research, greater vocabulary knowledge has been associated with advantages in language processing. In this study, we examined the relationship between individual differences in vocabulary and language processing performance more closely by (i) using a battery of vocabulary tests instead of just one test, and (ii) testing not only university students (Experiment 1) but young adults from a broader range of educational backgrounds (Experiment 2). Five vocabulary tests were developed, including multiple-choice and open antonym and synonym tests and a definition test, and administered together with two established measures of vocabulary. Language processing performance was measured using a lexical decision task. In Experiment 1, vocabulary and word frequency were found to predict word recognition speed while we did not observe an interaction between the effects. In Experiment 2, word recognition performance was predicted by word frequency and the interaction between word frequency and vocabulary, with high-vocabulary individuals showing smaller frequency effects. While overall the individual vocabulary tests were correlated and showed similar relationships with language processing as compared to a composite measure of all tests, they appeared to share less variance in Experiment 2 than in Experiment 1. Implications of our findings concerning the assessment of vocabulary size in individual differences studies and the investigation of individuals from more varied backgrounds are discussed.

## Introduction

Knowing the words of the language is undeniably an important part of a speaker's command of their language. Due to differences in life-experience, interests, and skills adults are likely to differ considerably in the structure and size of their native language vocabularies (Brysbaert et al., 2016b). Differences in lexical knowledge should lead to much variation in speakers' and listeners' ability to use language in spoken and written communication. However, only a few studies have investigated the actual role of adult speakers' vocabulary knowledge in language processing (e.g., Federmeier et al., 2002; Rodriguez-Aranda and Jakobsen, 2011; Yap et al., 2012; Banks et al., 2015). The present study builds upon this early work and investigates the relationship between individual differences in vocabulary size and language processing performance more comprehensively in two respects: by assessing vocabulary knowledge in a whole battery of tests rather than a single test, and by including not only university students, the typical participants in psycholinguistic studies, but also young adults in vocational colleges in the investigation, thereby testing samples that differ substantially in the formal schooling received and likely in the exposure to print.

Most studies that have considered the relationship between vocabulary size and language processing have found beneficial effects of increasing vocabulary size on language processing performance. Studies involving healthy older adults have shown, for instance, that having a larger vocabulary was beneficial for accurate spoken word recognition (Janse and Jesse, 2014) and the use of predictive information in spoken contexts (Federmeier et al., 2002). In young adults, an increase in vocabulary size was associated with better listening comprehension (Andringa et al., 2012). Large vocabulary size was also found to be linked to better speech recognition in suboptimal conditions (Bent et al., 2016). Furthermore, Yap et al. (2012) found higher vocabulary scores to be associated with more accurate and faster word recognition in lexical decision and speeded pronunciation tasks. In addition, increased vocabulary knowledge has been found to be associated with faster responses in picture naming (Rodriguez-Aranda and Jakobsen, 2011) and in verbal fluency tasks. In these latter tasks, participants are given 1 or 2 minutes to produce as many words as possible that belong to a given semantic category or start with a given letter (Rodriguez-Aranda and Jakobsen, 2011; Unsworth et al., 2011; Shao et al., 2014). In a nutshell, better vocabulary knowledge (i.e., knowing more words) has been associated with advantages in various language comprehension and production tasks. This is somewhat counterintuitive as one might expect that retrieving lexical items from a larger vocabulary would be slower than retrieval from a smaller lexicon because more lexical items might compete for selection as the lexicon becomes larger or denser (Diependaele et al., 2013). Contrary to this expectation, individuals with larger vocabularies appear to be able to access their knowledge faster than individuals with smaller vocabularies.

In addition to faster responses in language processing tasks, previous research has found smaller word frequency effects for speakers and readers with larger vocabularies (Yap et al., 2009; Diependaele et al., 2013) or more reading experience (Chateau and Jared, 2000; Kuperman and Van Dyke, 2013). Diependaele et al. (2013), for instance, reanalyzed data from an earlier visual word recognition study (Lemhöfer et al., 2008) and found larger vocabularies to be associated with smaller effects of word frequency. Similarly, Brysbaert et al. (2016a) observed that individuals with higher vocabulary scores were not only faster to make lexical decisions but also showed smaller effects of word frequency on their response speed. In both studies, this frequency by skill interaction was taken to be indicative of differences in entrenchment of words in smaller vs. larger vocabularies. Thus, in individuals with larger vocabularies lexical representations are assumed to be more robust or distinct, enabling faster processing, as compared to individuals with smaller vocabularies. According to this lexical entrenchment hypothesis the frequency by skill interaction is due to differences in exposure to language, especially to written language, which has a lower type-token ratio than spoken language (Brysbaert et al., 2016a). It is assumed that amount of exposure has a particularly strong impact on the representations of low-frequency words (Kuperman and Van Dyke, 2013). As a result, the lexicon of individuals with limited language exposure and therefore weaker word knowledge is hypothesized to show a stronger frequency difference between high- and low-frequency words.

A similar argument has been put forward by Yap et al. (2009). They also hypothesized that better vocabulary knowledge leads to overall increased precision and stability of lexical representations. This proposal was based on the observation that vocabulary knowledge affected the joint effects of word frequency and associative priming. In a lexical decision task, participants with poorer vocabulary knowledge showed stronger associative priming effects for low-frequency than for high-frequency words, whereas individuals with better vocabulary scores exhibited equally strong priming effects for both types of words. This suggests that the lexical representations in readers with larger vocabularies are equally strong for low- and high-frequency words so that all words can be processed equally fluently. The lexical representations in low-vocabulary individuals, by contrast, show considerable differences in strength or robustness depending on word frequency, which is reflected in stronger priming effects for low-frequency words (Yap et al., 2009).

To sum up, based on the observation that word frequency effects on word recognition are smaller in high-vocabulary than in low-vocabulary individuals, structural differences between the representations in vocabularies of varying sizes have been suggested. Researchers have used different terms to refer to this idea: Representations in individuals with better word knowledge or more experience with language have been proposed to be more robust, entrenched, precise, or higher in lexical quality making lexical access faster and less prone to effects of word frequency (Perfetti and Hart, 2001; Yap et al., 2009; Diependaele et al., 2013; Van Dyke et al., 2014).

While the aforementioned studies implicate a role of vocabulary size in comprehension and production tasks, most of them did not focus on vocabulary specifically. This may be the reason why usually only a single vocabulary test was employed to assess vocabulary size. It has been argued though that a complex construct, such as vocabulary size, cannot be measured using a single test (e.g., Bowles and Salthouse, 2008; De Bruin et al., 2017). This is because a person's performance on a test always depends on multiple factors, such as their vocabulary knowledge, world knowledge, guessing strategies, and attention. Bowles and Salthouse therefore recommended the use of a variety of tests of vocabulary size, especially in studies where vocabulary knowledge is the focus of interest.

Additionally, the majority of established vocabulary tests are multiple-choice tests but it is unclear whether this is the best way of assessing word knowledge. Gyllstad et al. (2015) compared second language learners' performance on a multiple-choice vocabulary test and an open interview-based test of vocabulary knowledge. They found that the multiple-choice test overestimated participants' vocabulary size compared to the open interview test, presumably because participants could use guessing or elimination strategies in the multiple-choice test. Although that study concerned second language learners, the argument can be extended to the assessment of native speakers. Thus, the findings reported by Gyllstad et al. further support the idea that multiple tests and test formats are required to obtain a valid indicator of vocabulary size.

Comprehensive investigations of individual differences in vocabulary, assessed using various different types of vocabulary tests, and their relationship with language processing performance in healthy adult native speakers are lacking so far. An important goal of the present study was to explore whether using multiple tests of vocabulary is indeed beneficial for predicting performance in a standard linguistic processing task, lexical decision. To this end Dutch participants completed seven vocabulary tests. Two of them were the established Dutch version of the Peabody Picture Vocabulary Test (PPVT-III NL; Schlichting, 2005) and Andringa et al.'s (2012) receptive multiple-choice test. In addition, five new tests of different types and formats were developed. These tests were a definition test, multiple-choice antonym and synonym tests, and open antonym and synonym tests. Thus, we took into account the idea that various test formats, i.e., multiple-choice and open tests, and asking the participants to perform different tasks, i.e., providing for example an antonym or synonym of a target, are needed to obtain a valid indicator of vocabulary size. The choice of the test types and formats was based on Henriksen's (1999) proposal that there are three dimensions of vocabulary development. The knowledge of words, which varies along a continuum from partial to precise, was addressed using the definition test and the various multiple-choice measures. Secondly, a deeper knowledge of the meaning of words and their relations to other words was assessed using the antonym and synonym tests. Finally, the distinction between receptive and productive vocabulary knowledge was taken into account by using open tests in addition to multiple-choice tests. Furthermore, we were inspired by the way vocabulary was assessed in earlier studies. The format of the multiple-choice synonym test was identical to widely used measures of vocabulary tests, such as the Shipley Vocabulary test (Shipley, 1946). The antonym test only differed from the synonym test in that participants were asked to select a word that had the opposite meaning as the target instead of the same meaning. The open tests should measure the same types of knowledge, but addressed concerns about mainly using multiple-choice tests to assess vocabulary knowledge (Gyllstad et al., 2015). The test items covered a large range of word frequencies to make sure that they measured sufficient variability in vocabulary.

In addition to completing the battery of vocabulary test, participants carried out a visual lexical decision task to measure the speed of word recognition (e.g., Balota et al., 2007; Keuleers et al., 2012; Brysbaert et al., 2016a). On each trial of the lexical decision task, participants are presented with a string of letters and decide whether or not it is an existing word in a given language. Two classic findings are the effects of lexicality and frequency. Responses for words are usually faster than responses for nonwords, and more frequent words elicit faster responses than less frequent words (e.g., Yap et al., 2012; Kuperman and Van Dyke, 2013; Keuleers et al., 2015).

We tested two hypotheses: (1) that better vocabulary knowledge would be associated with better performance (faster responses and/or lower error rates) in the lexical decision task, and (2) that better vocabulary knowledge would be associated with weaker word frequency effects in the lexical decision task. Thus, we tested the lexical entrenchment account, which predicts an interaction between participants' vocabulary scores and the word frequency effect (e.g., Diependaele et al., 2013). Further research questions concerned the vocabulary measures. Important issues were whether we would see this relationship with lexical processing for each of the vocabulary tests individually, and whether a composite score based on performance on all tests would constitute a better predictor of performance in the lexical decision task than any of the individual tests.

Experiment 1 was carried out with the “standard” participants for psycholinguistic studies, namely university students. Experiment 2 was very similar but was conducted with a roughly age-matched sample of young adults attending vocational colleges. Within this group, one would expect to find a much broader range of linguistic skills than in a group of university students. Replicating the study with this group was important for two reasons. First, it provided important information about the battery of vocabulary tests, specifically about its suitability for assessing vocabulary in adults with a broader linguistic ability range than typically seen in university students and about the correlations among the tests. This information should be useful for further research as well as for practical purposes. Second, it allowed us to assess whether the results of Experiment 1 concerning the relationship between vocabulary size and lexical decision performance would be replicated in this broader sample. Though this is rarely done, including persons who are not university students in psycholinguistic studies is evidently important for ascertaining that processing theories based on the results of these studies hold for adults in general, rather than just for university students.

## Experiment 1

### Method

#### Participants

A total of 75 young adults (57 females) aged between 18 and 34 years (*M* = 21.9; *SD* = 3.7) gave informed written consent to participate in this study^1^. All participants were completing their studies at the Radboud University Nijmegen or the Hogeschool van Arnhem en Nijmegen at the time of testing or had recently graduated. Forty-seven of the participants indicated that they were fluent in English. Seven of them were in addition to that also fluent in German (*N* = 3), French (*N* = 2), Chinese (*N* = 1), or Spanish (*N* = 1). All participants were recruited using the participant database of the Max Planck Institute for Psycholinguistics and were paid 12 Euros for their participation. Ethical approval was granted by the Faculty of Social Sciences of the Radboud University Nijmegen.

#### Materials and design

All participants completed a battery of seven vocabulary tests and a lexical decision task. Two of the vocabulary tests were established measures of vocabulary knowledge, namely Andringa et al.'s (2012) receptive multiple-choice test and the Peabody Picture Vocabulary Test (PPVT-III NL; Schlichting, 2005). The other five tests were newly developed.

##### Receptive multiple-choice test

This multiple-choice test was developed by Andringa et al. (2012). Participants were presented with target words, such as *mentaliteit* (mentality) or *tentatief* (tentative), embedded in different neutral carrier sentences. Each sentence was presented along with five answer options, one of which was a description of the target word and one was *Ik weet het echt niet* (I really don't know). For example, the target word *mentaliteit* (mentality) was presented with the answer options *tafel* (table), *persoon* (person), *manier van denken* (way of thinking), and *sfeer* (atmosphere; see Appendix A for examples and the Supplementary Materials for all materials of this receptive multiple-choice test and the five new vocabulary measures). The target words covered a large range of word frequencies between 0 and 31.28 counts per million in the SUBTLEX corpus (*M* = 1.87; *SD* = 5.07; Keuleers et al., 2010).

The test consists of 60 target sentences. In the present study the first sentence was used as a practice item so that the test comprised a total of 59 questions. The sequence of items and the positions of the response alternatives for each item were the same as in Andringa et al.'s (2012) test.

##### Peabody picture vocabulary test (PPVT)

Every trial in the Dutch version of the PPVT-III (Schlichting, 2005) consists of a spoken target word and a set of four pictures. Participants were instructed to choose the picture that corresponded to the word they heard. The target word frequency ranged from 0 to 29.13 counts per million in the SUBTLEX corpus (*M* = 1.86; *SD* = 4.60; Keuleers et al., 2010).

The stimuli in the PPVT are organized in blocks of 12 words but the number and order of blocks varied depending on the participant's performance. Each participant started with the same first block of 12 items. Depending on the number of mistakes made, the following block was comprised of either easier or more difficult words. The same held true for all subsequent blocks of stimuli. Thus, individuals with poor word knowledge might complete five blocks of 12 words each, while others might complete the maximum of eight blocks of stimuli. All target words had been spoken by a female native speaker of Dutch and recorded.

##### Definition test

In this test, participants were presented with 20 definitions of words from four different semantic categories (animal, profession, body part, instrument/object). The task was to write down the word that corresponded to the definition. All definitions were taken from a definition naming experiment by La Heij et al. (1993). The frequencies of the correct responses ranged between 0.02 and 244.07 occurrences per million (*M* = 39.01, *SD* = 63.63) in the subtitle corpus SUBTLEX-NL corpus (Keuleers et al., 2010). The correct responses displayed *z*-transformed prevalence values between 1.99 and 3.41 (*M* = 2.93, *SD* = 0.44). Prevalence is a measurement of how many people in a group of speakers of a language, in this case Dutch, know a given word (Keuleers et al., 2015). Following Keuleers and colleagues, words with a prevalence value of 1 or above are known by at least 85% of Dutch native speakers in the Netherlands. The order of items within this and the following tests was the same for every participant and pseudo-randomized such that low- and high-frequency test items were well distributed.

##### Multiple-choice antonym test

The multiple-choice antonym test included 25 test items, which were presented without carrier sentences along with five single-word answer alternatives. Some of the target words were taken from the Toets Gesproken Nederlands (TGN), a Dutch language test used to assess language for immigration requirements (Kerkhoff et al., 2005).

The test items in the multiple-choice antonym test represented a large frequency range with between 0 and 3838.54 counts per million (*M* = 200.16, *SD* = 764.93) in the subtitle corpus SUBTLEX-NL (Keuleers et al., 2010) and *z*-transformed prevalence values of between −1.73 and 3.37 (*M* = 2.37, *SD* = 1.22).

##### Open antonym test

Just as the multiple-choice test, the open antonym test included 25 test items, which were presented individually. Participants were instructed to write down an antonym for each word. Some of the target words were also taken from the TGN (Kerkhoff et al., 2005). The test items represented a frequency range between 0 and 60.69 counts per million in the SUBTLEX-NL corpus (*M* = 9.09, *SD* = 13.14). The prevalence values of the target words ranged from 1.03 to 3.32 (*M* = 2.49, *SD* = 0.68).

##### Multiple-choice synonym test

The multiple-choice synonym test was structurally identical to the multiple-choice antonym test, the only difference being that participants were asked to select a word that had the same meaning as or was interchangeable with the target. It consisted of 25 test items, which were presented along with five single-word answer alternatives. The multiple-choice synonym test was based on a part of the Groningen Intelligence Test (Luteijn and van der Ploeg, 1983). This measurement consists of 20 test items, which are presented along with five answer options each. The majority of these words have very low frequencies in the SUBTLEX-NL corpus. In order to adapt the test for the present purposes and make the final test scores comparable to the 25-item antonym test, five new medium to high-frequency test words were added.

The word frequencies of the test items ranged from 0 to 48.05 per million (*M* = 4.85, *SD* = 11.02) in the SUBTLEX-NL corpus. The words' prevalence values ranged between −0.64 to 3.35 (*M* = 1.77, *SD* = 1.11).

##### Open synonym test

The open synonym test was structurally identical to its antonym counterpart. It comprised 25 test items which were presented individually and without carrier sentences. The construction of this open synonym test was inspired by the English version of the Mill Hill Vocabulary Scale (Raven et al., 1998). The target words had frequency values between 0 and 36.25 per million (*M* = 4.62, *SD* = 9.33) and their prevalence values ranged between −0.59 and 3.32 (*M* = 1.88, *SD* = 1.06). Each word appeared only once as a target item across all vocabulary tests.

##### Lexical decision task

Ninety word and 90 nonword stimuli were included in the lexical decision task. The words covered a broad word frequency range from 0.02 to 89.92 (*M* = 9.69, *SD* = 16.38) occurrences per million in the SUBTLEX corpus (Keuleers et al., 2010). All words were uninflected and were not homophonous with other Dutch words. The words' prevalence values were at least 2.89, indicating that all words were known by at least 98% of Dutch speakers (Keuleers et al., 2015).

The nonwords were created using the program Wuggy (Keuleers and Brysbaert, 2010), which generates nonwords based on real words. Each of the nonwords corresponded to one real word on the list. All nonwords differed from their real word counterparts in a letter, a sound, or an entire syllable, while being pronounceable but not homophonous with an existing Dutch word. Four lists including all 180 items each were created. The order of stimuli within each list was fixed. Not more than three consecutive trials belonged to the same experimental condition. The four stimuli lists were counterbalanced across participants.

#### Apparatus

All tasks were presented on a 17-inch screen (Iiyama LM704UT) using Presentation software (version 16.5, www.neurobs.com) or as an online questionnaire using LimeSurvey (www.limesurvey.org). The auditory stimuli in the PPVT were presented using HD 280 Sennheiser headphones.

#### Procedure

The participants were tested individually in experiment rooms at the Max Planck Institute for Psycholinguistics. Everyone completed the vocabulary tests in the same order, namely definition test, Andringa et al.'s (2012) test, multiple-choice antonym test, open antonym test, multiple-choice synonym test, open synonym test, and PPVT. The vocabulary tests were self-paced and participants were instructed to answer as accurately as possible without thinking about single test items for too long. Before each of the tests started, instructions were presented on the screen, along with example items. In total, the vocabulary test battery took between 35 and 45 min. The lexical decision task was completed in a separate test session.

##### Receptive multiple-choice test

The target words were presented in neutral carrier sentences and were marked with two asterisks. The question and answer options were written in 25-point Arial font and stayed on the screen until the participant had selected an answer by pressing one of the number buttons 1 to 4 on the keyboard. Answering a question initiated the presentation of the following test item.

##### Peabody picture vocabulary test

Participants were presented with a spoken stimulus and four pictures on the screen and were instructed to select one of four pictures that corresponded to the word they heard by pressing one of the number buttons one to four on the keyboard. The target word was repeated until one of four response buttons were pressed, which initiated the presentation of the next stimulus.

##### New vocabulary tests

In the definition and open antonym and synonym tests, participants were asked to type in the correct answer using the keyboard. For the definition test, the answer would be the animal, profession, body part, or object corresponding to the definition. In the open antonym and synonym tests, participants were required to type in a word that is an antonym or synonym of the test word. Participants could skip an item and proceed to the next when they did not know the answer.

In the multiple-choice antonym and synonym tests, participants were given five answer alternatives and instructed to select the word that had the opposite (antonym) or same (synonym) meaning as the target. Answering a question initiated the presentation of the following test item. All text was written in 25-point Arial font.

##### Lexical decision task

The experiment was divided into two parts, consisting of 90 stimuli each. Between the two blocks, participants could take a short break. Each trial started with a fixation cross, which was shown in the center of the screen. After 500 ms, it was replaced by a word or nonword written in 24-point Arial font. The stimuli stayed on the screen for 3 s or until a response button was pressed. Half of the participants were instructed to press the “Z” button on the keyboard if the string of letters on the screen was a word and “M” if it was a nonword; for the other half the instruction was reversed. Participants were instructed to respond as quickly and accurately as possible. Before the test phase began, participants were familiarized with the task in four practice trials.

#### Analyses

##### Vocabulary tests

Peabody scores were calculated based on the total number of items participants responded to and the number of errors they made. These raw scores were then transformed into standardized scores, called *woordbegripquotient* (word comprehension score, WBQ), which was used for all further analyses (Schlichting, 2005). One point was given for each correct answer in all other multiple-choice and open tests. Some exceptions applied to the definition as well as the open antonym and synonym tests. If a participant demonstrated knowledge of the word or concept without producing an actual antonym [e.g., writing *stil* (silent) instead of *stilte* (silence) as antonym for *lawaai* (noise)] or if the answer was misspelled, they received 0.5 points for that answer. Three native speakers of Dutch with backgrounds in linguistics or psycholinguistics first independently categorized all answers and then discussed any cases where they disagreed. This always resulted in a judgment supported by all of them. For some targets several responses were categorized as correct. For example, in the case of the definition test item *Iemand die werkt met patiënten* (*Someone who works with patients*), the responses *dokter*, and *arts* (both *doctor*) as well as *verpleegster* (*nurse*) were considered correct. The fact that different concepts met some of the definitions may be considered a weakness of the definition test. In the open antonym and synonym tests, synonyms were considered correct, such as *rust/stilte* (silence) as antonyms of *lawaai* (noise).

The relationships between the vocabulary test scores were analyzed using bivariate correlation analyses. In addition we conducted a Principal Component Analysis (PCA) in SPSS, which can be used to identify a small number of components that account for the variability in a larger number of original measures, in this case seven vocabulary tests. The goal of a PCA is data reduction in cases where the measures originally included in the study are to be reduced to a subset without losing information (DeCoster, 1998). We ran a PCA assuming two components for that exact reason, namely reduction of the number of vocabulary scores as predictors in the individual differences analyses to one measure or two measures (reflecting a distinction between multiple-choice and open tests).

##### Lexical decision task

In the lexical decision task, we measured response times (RTs) and accuracy. Responses were excluded from the analyses if they exceeded a participant's mean RT by more than three standard deviations (SDs) or were shorter than 250 ms.

Accuracy was investigated using mixed logit models employing the glmer function from the package lme4 (version 1.1.12; Bates et al., 2015) in R (R Core Team, 2016). The first model on words and nonwords included a intercept, a fixed effect for lexicality (word vs. nonword) as well as random intercepts for both item and participant. Additionally we modeled per-participant random slope adjustments to the lexicality effect. Secondly, a model on words alone was run including a intercept, a fixed effect for frequency, and random intercepts for both items and participants. Per-participant random slope adjustments to the frequency effect were also included.

RTs were log-transformed and analyzed in linear mixed-effects models using the lmer function of the lme4 package (version 1.1.12; Bates et al., 2015) in R (R Core Team, 2016). RTs for correct responses to words vs. nonwords and to words alone were analyzed. The first mixed model on RTs for correct responses to words and nonwords included a intercept, a fixed effect for lexicality, and by-participant and by-item adjustments to the intercept (random intercepts). Additionally, by-participant random slope adjustments to the lexicality effect were included. The predictor lexicality was sum-to-zero contrast coded (nonword = 1; word = −1).

The second model on correct responses to words only included an intercept, and a fixed effect for the log-transformed continuous variable word frequency. Furthermore, by-participant and by-trial random adjustments to the intercept (random intercepts) and by-participant random adjustments to the frequency slope were modeled (random slope). All possible correlations between the random effects were included. Hence, we followed Barr et al. (2013) using a maximal random effects structure. *P*-values were determined using the normal approximation, i.e., using the *t*-value as *z*-value.

In order to examine the effects of individual differences in vocabulary knowledge on lexical decision task performance, the models of RTs and accuracy for words were run with a vocabulary score as an additional predictor. Both models included an intercept, a fixed effect for the continuous variable word frequency, and by-participant and by-trial random adjustments to the intercept (random intercepts). In addition, by-participant random adjustments to the frequency slope were modeled (random slope). The model for accuracy did not converge with this maximum random effects structure. We therefore had to remove the random slopes from the model.

In order to explore how well the scores from each of the vocabulary tests predicted the speed and accuracy in the lexical decision tasks, we ran separate models for each score, yielding seven models predicting accuracy and seven models predicting speed. In addition, we used a composite measure of vocabulary described below. Based on the PCA on all vocabulary tests, which did not show a clear pattern distinguishing different types of tests from one another (see below), we decided to use a component score of vocabulary reflecting each participant's performance on all seven measures of word knowledge. For that purpose, regression-based factor scores were calculated for each participant using the PCA method in SPSS (DiStefano et al., 2009). Assuming only one underlying factor, each individual's loading on that factor based on their seven vocabulary test scores was calculated and included in the regression analyses. This allowed us to compare the individual vocabulary measures with a composite measure reflecting performance on the entire battery of tests. We calculated the conditional *R*^2^ in R using the function sem.model.fits from the piecewiseSEM package (version 1.2.1; Lefcheck, 2015). The conditional *R*^2^ describes the proportion of variance explained by both the fixed and random factors, for each model.

### Results

#### Vocabulary tests

Table 1 shows the vocabulary test scores, averaged across participants and Table 2 displays the correlations between the test scores. There were moderate to strong correlations between all test scores, indicating that the vocabulary measures assessed, to some extent, a shared underlying ability. The multiple-choice antonym test, which was easier than the other tests, was least strongly correlated with the other measures. The reliability measure Cronbach's α indicated that the test battery as a whole is reliable (α = 0.88). Dropping the multiple-choice antonym test would increase α (0.89) while leaving out one of the other tests would lead to a lower α.

**Table 1:** Test scores in seven vocabulary tests.

**Test**	***N***	**Minimum**	**Maximum**	**Mean**	***SD***
Andringa	75	25.0	58.0	40.01	6.47
Peabody	75	56.0	125.0	102.61	12.50
Definition test	75	12.0	20.0	16.41	2.04
Antonym MC	75	14.0	25.0	23.0	1.60
Antonym open	75	14.0	24.0	19.29	2.37
Synonym MC	75	11.0	24.0	17.68	2.87
Synonym open	75	5.50	22.0	10.70	2.86

**Table 2:** Correlations between the different vocabulary scores.

	**Definition**	**Andringa**	**Antonym MC**	**Antonym open**	**Synonym MC**	**Synonym open**
Andringa	0.62^**^					
Antonym MC	0.46^**^	0.36^**^				
Antonym open	0.73^**^	0.63^**^	0.40^**^			
Synonym MC	0.59^**^	0.62^**^	0.32^**^	0.58^**^		
Synonym open	0.68^**^	0.61^**^	0.38^**^	0.63^**^	0.56^**^	
Peabody	0.63^**^	0.57^**^	0.35^**^	0.67^**^	0.58^**^	0.59^**^

** *p < 0.001*.

A PCA assuming two components was run on *z*-transformed vocabulary scores. This was based on the assumption that two components might distinguish between multiple-choice and open tests. The first component had an eigenvalue of 4.35, the other component had an eigenvalue below 1. This first component explained 62.13% of the total variance. As shown in Table 3, Factor 1 loaded on all tests with only a slightly smaller loading for the multiple-choice antonym test. No distinction between productive and receptive vocabulary tests was found.

**Table 3:** Results of the Principal Component Analysis assuming two components.

**Vocabulary measure**	**Component**	
	**1**	**2**
Andringa	0.81	−0.13
Peabody	0.80	−0.14
Definition	0.86	0.04
Antonym MC	0.56	0.82
Antonym open	0.84	−0.07
Synonym MC	0.78	−0.22
Synonym open	0.82	−0.06
Eigenvalue	4.35	0.76
% Variance	62.13	10.89

#### Lexical decision task

Accuracy rates were overall high with 2.7% of all trials being false alarms and 1.6% being misses. RTs were trimmed per participant according to the above-mentioned criteria. 1.7% of trials were excluded as outliers. As typically found in lexical decision tasks, accuracy was higher for words than for nonwords (*z* = −4.56; *p* < 0.001) and participants made fewer errors with increasing word frequency (*z* = 7.36; *p* < 0.001). In addition, RTs for words were significantly faster than for nonwords (*t* = 10.74, *p* < 0.001; see Appendix B in Supplementary Material for a table showing averaged lexical decision RTs for all conditions). Finally, RTs for correct responses to words were significantly predicted by word frequency (*t* = −15.17; *p* < 0.001), with faster responses being associated with higher word frequency.

#### Individual differences

The main interest of the present study was the relation between individual differences in vocabulary and lexical processing. Results of mixed-effects models of both accuracy and speed in the lexical decision task are reported below. For this individual differences investigation, we focused on responses to word trials. We used Bonferroni adjusted alpha levels of 0.006 per test (0.05/8) because all models were run with the composite measure as well as each of the seven vocabulary tests as predictor.

##### Accuracy

Response accuracy for words was significantly predicted by word frequency (*z* = 7.33; *p* < 0.001), with lower error rates being associated with higher word frequency, but not by the composite vocabulary score (*z* = 1.50; *p* = 0.13). The interaction between word frequency and the composite vocabulary score was not significant (*z* = 0.08; *p* = 0.93).

The models using vocabulary scores from the individual tests as predictors of accuracy showed overall the same results (see Appendix D in Supplementary Material). Word frequency was a highly significant predictor of lexical decision accuracy in all seven models, whereas vocabulary was insignificant in five of the models. Only the scores from Andringa et al.'s (2012) measure and the open antonym test were significant predictors of lexical decision accuracy. For all models the conditional *R*^2^ was 0.31.

##### Reaction times

Lexical decision RTs on word trials were significantly predicted by log-transformed word frequency (*t* = −15.21; *p* < 0.001) and the composite vocabulary score (*t* = −3.10; *p* = 0.002). RTs decreased with increasing word frequency, as typically found in the lexical decision task, and individuals with higher vocabulary scores responded faster than individuals with weaker vocabulary knowledge. The interaction between the two main effects, word frequency and vocabulary score, was not significant (*t* = 1.23; *p* = 0.22; see Appendix C in Supplementary Material). The seven mixed-effects models, each including the scores from one of the individual tests and word frequency as predictors, confirmed this overall (see Appendix D in Supplementary Material). Vocabulary was a significant predictor of lexical decision RTs in all models with the exception of the model for the multiple-choice antonym test. Furthermore, the scores from the definition, open antonym, and open synonym tests were significant predictors of lexical decision RTs even after correcting the alpha level to 0.006; the scores from Andringa et al.'s (2012) test and the multiple-choice synonym and PPVT tests did not reach the corrected significance level^2^. The conditional *R*^2^ scores were similar for all models, with 0.45 for the models with the scores from Andringa et al.'s (2012) receptive multiple-choice test and the PPVT and 0.46 for all other models.

### Discussion

In Experiment 1, participants' vocabulary was assessed in a battery of seven tests, and their scores on the individual tests and a composite vocabulary score were related to their performance in a visual lexical decision task. An important goal of this study was to explore how strongly the vocabulary test scores would correlate with each other and whether a better prediction of lexical decision performance would be achieved using the composite score compared to individual test scores. The bivariate correlations were similarly strong for most pairs of vocabulary tests with the exception of the correlations involving the multiple-choice antonym test. As the average score and low SD indicate, this test was too easy for the group. The high correlations between the test scores indicate that all of them measure largely the same ability. We did not find higher correlations between tests using the same modality (production or comprehension) compared to tests using different modalities or between tests using the same format (multiple choice or open answer) compared to tests using different formats. However, the open tests were not interview-based measures; they were not fully open as they still provided the participants with word stimuli and specific tasks to perform on them, namely writing down antonyms or synonyms of the given words. This might be the reason why no distinction was found between multiple-choice and open tests, contrary to earlier findings (Gyllstad et al., 2015). The reliability measure Cronbach's α was relatively high for the test battery as a whole.

A PCA confirmed these conclusions. First, no distinction between multiple-choice and open tests in terms of two distinct components was found. Secondly, only the multiple-choice antonym test did not show a loading as high as the other tests on the first component and instead loaded highly on the second component. This is in line with the conclusions drawn from the descriptive, correlational, and reliability analyses indicating that the multiple-choice antonym test does not relate well to the other measures, presumably as it was much easier than the remaining tests. We acknowledge this difference in difficulty. It is probably related to the higher word frequencies of the test items in this test as compared to the other tests^3^. If the entire battery of vocabulary tests were to be used again, one might want to adjust the frequency ranges so that they are more similar across all tests.

The fact that the correlations between the vocabulary test scores were substantial but not perfect supports the view that the tests tapped a common skill as well as unique skills that were not shared between all tests (Bowles and Salthouse, 2008; De Bruin et al., 2017). The substantial correlations between the scores explain that the composite score did not predict the performance in the lexical decision task better than the individual test scores. In other words, for predicting performance on the lexical decision task, using one vocabulary test was as useful as using the battery of tests. Hence, based on the current results no specific measure of vocabulary can be recommended as being superior to the others. All tests appear to assess word knowledge equally well.

Turning to the results of the lexical decision task in more detail, the typical effects of lexicality and word frequency on accuracy and RTs were found, with more accurate and faster responses for words compared to nonwords, and for higher compared to lower frequency words. Weaker vocabulary scores were associated with slower RTs in the lexical decision task. This finding is consistent with findings of several earlier studies (Unsworth et al., 2011; Yap et al., 2012; Diependaele et al., 2013; Brysbaert et al., 2016a).

Deviating from previous studies (Chateau and Jared, 2000; Yap et al., 2009; Diependaele et al., 2013; Kuperman and Van Dyke, 2013), the size of the word frequency effect was independent of vocabulary size. In other words we did not find evidence for the frequency by skill interaction predicted on the basis of the lexical entrenchment hypothesis. Given that we obtained strong effects of word frequency on accuracy and RTs, it is unlikely that the frequency range covered by our materials was too small to allow us to detect the interaction. However, it has to be noticed that the word frequency range was smaller than the range in the materials used by Brysbaert et al. (2016a), with a minimum SUBTLEX frequency of 0.12 and a maximum of 501.33 (*M* = 18.73; see Adelman et al., 2014, for the materials used in Brysbaert et al., 2016a).

In sum, we found that vocabulary size was related to performance in the lexical decision task, with larger vocabulary being associated with better performance in the lexical decision task, but we did not find the predicted frequency by skill interaction. These results do not support the lexical entrenchment hypothesis, according to which larger vocabularies are characterized by smaller differences in the quality of the representations of words differing in frequency. One account of these findings is that our sample, university students, was too homogeneous in their lexical skills to display this interaction. Dutch university students have similar educational backgrounds (usually at least 13 years of formal schooling) and they are probably also rather homogeneous in the amount and type of language exposure they get by reading, attending lectures, and so forth. Hence, all participants were presumably highly proficient users of their native language. Although there was variation in vocabulary scores as well as in lexical decision RTs this variation may not have been strong enough to allow us to detect a frequency by skill interaction.

## Experiment 2

As indicated above, we might not have been able to detect a frequency by skill interaction in Experiment 1 because the sample was too homogeneous in their linguistic skills. To assess this suggestion we repeated the study with a group of vocational college students. Vocational education in the Netherlands is subdivided into four levels, with level one courses providing the most basic general and vocational skills and graduation from a level four program giving access to higher education (university) programs. Many but not all students progress from a lower to a higher level of education. Thus, this group of students is rather varied in the amount of formal education they have received and should display a considerably broader range of vocabulary knowledge than university students.

In addition to following up on the lack of the frequency by skill interaction in Experiment 1, the second experiment allowed us to assess how suitable the vocabulary tests were for assessing vocabulary size in young adults who are not university students, and to examine again how the scores for different tests were related to each other.

In this experiment, we used the expected variability in language skills in the chosen sample to assess a specific hypothesis. However, information about language processing in persons who are not university students is evidently important in its own right. This information is needed to ascertain that psycholinguistic theories that have been almost entirely based on studies involving young academics apply to listeners and speakers outside of this group and it is of obvious societal importance.

### Method

#### Participants

A total of 231 young adults gave informed written consent to participate in this study. All of them were students at vocational colleges in the Netherlands (ROC Nijmegen, ROC Tilburg, ROC Midden Nederland). All participants were recruited through the teachers at their colleges. Participation was voluntary and not part of compulsory classes. In some cases, the schools were paid an expense allowance of 10 Euros per participant to spend on teaching materials; in other schools, participants were paid 10 Euros each for their participation. Ethical approval was granted by the Faculty of Social Sciences of the Radboud University Nijmegen.

The results obtained from 57 individuals were excluded due to failure to perform one or several of the tasks correctly. Performance on the PPVT or the lexical decision task was in most cases the reason for excluding a participant. Data of 174 participants (92 females) aged between 18 and 32 years (*M* = 20.3; *SD* = 2.7) were left for further analyses. Table 4 shows the distribution of participants across the different levels of vocational education. A more even spread across the levels would certainly have been desirable, but could not be attained due to timetabling constraints in the colleges. Forty-eight of the 174 participants indicated that they spoke English fluently, most of which were in level 4 (*N* = 31; level 3: *N* = 13; level 2: *N* = 2; level 1: *N* = 1). One of them was also fluent in German and six more people were fluent in Papiamento, Polish, Russian, Croatian, and Turkish (*N* = 2).

**Table 4:** Participant characteristics by group and level of education in Experiment 2.

**Level**	**Frequency**		
	**female**	**male**	**total**
1	3	−	3
2	14	−	14
3	24	27	51
4	50	56	106
Total	92	83	174

#### Materials and design

The materials of the lexical decision task were the same as in Experiment 1, whereas the vocabulary test materials were slightly different. The open synonym test was not administered because the scores achieved by the university students indicated that it would probably be very challenging and potentially frustrating for the vocational college students without providing valuable additional insights^4^. Furthermore, five additional high-frequency filler words were added to the multiple-choice antonym and synonym tests as well as the open antonym test. This was done in order to increase the number of relatively easy items and keep participants motivated throughout the test. These filler items were excluded from the final test score.

#### Apparatus

All tasks were administered using 14-inch HP laptops (Probook 640 G1) and Panasonic RP-HT030 headphones. All tests were implemented using Presentation software (version 16.5, www.neurobs.com).

#### Procedure

Participants were tested in groups of 9–30 students in their classrooms. All of them completed the tasks in the same order, with the vocabulary tests first followed by the LDT. The vocabulary measures were administered in the same order as in Experiment 1, except that the open synonym test was omitted. The procedure for the vocabulary tests was the same as in Experiment 1.

The procedure for the lexical decision task was slightly altered. Firstly, a pilot study with 20 vocational college students showed that presenting the stimuli for 3 s was too short. In this pilot study, 11 out of 20 individuals had error rates of 30% or higher, seven of them had error rates above 50%. Therefore, the presentation time was increased to 5 s. Secondly, the response buttons were kept constant across participants, with “M” to be pressed for words and “Z” for nonwords. This was done to facilitate administering the task in a group setting. Note that for practical reasons, the vocabulary tests and lexical decision task were administered in a single session, whereas university students were tested in two sessions.

### Results

#### Vocabulary tests

The responses in the vocabulary tests were scored and analyzed as described above. The mean vocabulary test scores per test are shown in Table 5.

**Table 5:** The distribution of test scores in all six vocabulary tests in Experiment 2.

**Test**	***N***	**Minimum**	**Maximum**	**Mean**	***SD***
Andringa	174	15.0	50.0	33.41	6.03
Peabody	174	55.0	115.0	87.85	10.0
Definition test	174	6.0	20.0	14.67	2.11
Antonym MC	174	8.0	25.0	20.79	2.64
Antonym open	174	7.0	21.5	14.85	2.36
Synonym MC	174	3.0	21.0	12.81	3.19

Bivariate correlation coefficients between the vocabulary tests are displayed in Table 6. All measures were moderately, but significantly correlated with one another and thus appear to capture a shared underlying variable. The reliability measure Cronbach's α indicated that the test battery as a whole is reliable (α = 0.80). Dropping one of the tests would lead to a lower α, hence lower reliability of the test battery.

**Table 6:** Correlations between the different vocabulary scores for the group of vocational college students.

	**Definition**	**Andringa**	**Antonym MC**	**Antonym open**	**Synonym MC**
Andringa	0.46^**^				
Antonym MC	0.39^**^	0.57^**^			
Antonym open	0.20^**^	0.38^**^	0.44^**^		
Synonym MC	0.36^**^	0.56^**^	0.43^**^	0.40^**^	
Peabody	0.32^**^	0.35^**^	0.42^**^	0.34^**^	0.40^**^

** *p < 0.001*.

A PCA assuming two components was run on *z*-transformed vocabulary scores. Only the first component had an eigenvalue >1 and it explained 50.39% of the total variance. Factor 1 loaded on all vocabulary tests (see Table 7). Again, no clear picture about a distinction between productive and receptive vocabulary tests was obtained.

**Table 7:** Results of the Principal Component Analysis assuming two components.

**Vocabulary measure**	**Component**	
	**1**	**2**
Andringa	0.80	−0.17
Peabody	0.65	0.17
Definition	0.63	−0.64
Antonym MC	0.77	0.05
Antonym open	0.64	0.59
Synonym MC	0.75	0.01
Eigenvalue	3.02	0.81
% Variance	50.39	13.56

#### Lexical decision task

Accuracy rates were lower than usually observed in lexical decision tasks and as in Experiment 1, with 11.6% of all trials being false alarms and 2.3% misses. RTs were trimmed by excluding all responses that exceeded a participant's mean by 3 SD or were lower than 250 ms. Following these criteria, 3% of data were excluded as outliers. Accuracy was higher for words than for nonwords (*z* = −20.61; *p* < 0.001), and participants made fewer errors with increasing word frequency (*z* = 10.98; *p* < 0.001). RTs were faster for words than for nonwords (*t* = 19.18; *p* < 0.001; see Appendix B in Supplementary Material). Finally, RTs for correct responses to words increased with decreasing word frequency (*t* = −14.29; *p* < 0.001).

#### Individual differences

##### Accuracy

We used Bonferroni adjusted alpha levels of 0.007 per test (0.05/7). Response accuracy in the lexical decision task was predicted by word frequency (*z* = 8.72, *p* < 0.001) and the composite vocabulary score (*z* = 4.46; *p* < 0.001). The interaction between these main effects was not significant (*z* = −0.89; *p* = 0.37). Similar results were obtained in the mixed effects models including the scores from the individual vocabulary tests as predictor (see Appendix F in Supplementary Material). Only the model using the PPVT scores as one of the predictors along with word frequency did not show a significant main effect of vocabulary score (*z* = 1.32; *p* = 0.19) but only of word frequency (*z* = 8.0; *p* < 0.001). The interaction was not significant (*z* = −0.97; *p* = 0.33), just as in the model including the composite vocabulary score. The model including the multiple-choice synonym test scores as predictor of lexical decision accuracy on word trials did not converge, even after excluding random slopes and the random intercept for item. All other models showed the same patterns of relationship between vocabulary score, word frequency, and lexical decision accuracy. The conditional *R*^2^ was 0.39 for all models, except for the models including the multiple-choice antonym (*R*^2^ = 0.40) and the synonym multiple-choice tests (*R*^2^ = 0.38).

##### Reaction times

There was a significant main effect of word frequency (*t* = −14.93; *p* < 0.001) but not of the composite measure of vocabulary (*t* = 0.91; *p* = 0.36) on log-transformed RTs. Importantly, the interaction between word frequency and the composite vocabulary score was significant (*t* = 4.01; *p* < 0.001), with a stronger word frequency effect for individuals with poorer word knowledge (see Appendix E in Supplementary Material). Most of the models including one of the individual vocabulary scores as predictors of lexical decision RTs yielded similar results (see Appendix F in Supplementary Material for the results of all seven models). In the model using the open antonym score and word frequency as predictors, the latter was highly significant (*t* = −12.22; *p* < 0.001); however, neither vocabulary (*t* = −1.40; *p* = 0.16) nor the interaction between word frequency and vocabulary (*t* = 1.33; *p* = 0.18) were significant predictors of RT. The scores from the multiple-choice synonym and antonym tests did not show significant main effects on lexical decision RTs after correcting the significance level for multiple tests. The conditional *R*^2^ was 0.27 for all models.

### Discussion

Experiment 2 was a near replication of Experiment 1 with a new sample, students of vocational colleges rather than university students. Our goals were, first, to evaluate the usefulness of the battery of vocabulary tests for assessing vocabulary size in this group, and, second, to determine whether and how vocabulary size affected the performance in the lexical decision task.

Though there was considerable overlap between the two groups, the average vocabulary scores obtained by vocational college students were lower and the scores were more variable than the scores obtained by university students. This was expected given that individuals from all levels of vocational education were tested, presumably covering a large range of abilities. The scores from all vocabulary measures correlated with each other, but, as Tables 2 and 6 show, the correlations were not as strong as seen for the university students. In other words, in the group of vocational college students the vocabulary tests captured less shared variance than in the group of university students. In line with that, the PCA showed a smaller percentage of variance explained by the first component as well as weaker loadings of it on all vocabulary measures. However, again no distinction between open and multiple-choice tests was found. In addition, the composite vocabulary score, which captures what is shared between all tests, did not predict lexical decision speed better than the individual vocabulary scores. At first sight, the results of the analyses using the individual vocabulary scores as predictors of lexical decision performance were more varied in Experiment 2 than in Experiment 1, perhaps because in the latter group, the vocabulary tests were more strongly correlated and shared more variance. However, the conditional *R*^2^ values indicate that all models, each including a different measure of vocabulary as predictor, explained approximately the same amount of variance lexical decision accuracy and RTs.

Notably, just as in Experiment 1, the mean vocabulary scores indicated that the multiple-choice antonym test was easier than the multiple-choice synonym and open antonym tests. We acknowledge this difference in difficulty, which was observed in both experiments. As noted, in future research one might want to adjust the frequency ranges so that they are more similar across all tests.

Analyses of the lexical decision data showed the expected lexicality and word frequency effects: As in earlier studies, error rates and RTs were lower for words as opposed to nonwords and for high- as compared to low-frequency words (e.g., Yap et al., 2012; Kuperman and Van Dyke, 2013; Keuleers et al., 2015). Error rates were, however, considerably higher than in Experiment 1 and typically reported in the literature, and the RTs were much longer (by 170 ms for word decisions and 300 ms for nonword decisions) and more variable (see Appendix B in Supplementary Material). It is impossible to say how this substantial group difference arose. All words had high prevalence values, indicating that at least 98% of Dutch speakers knew them. Thus, participants could be assumed to know most of the words. Some participants may have been rather poor readers and may have taken long and variable times to decode the items. These participants may have fared better with auditory presentation of the words. It is also possible that the vocational college students considered their responses, in particular the nonword decisions, more carefully than the university students, perhaps because they were less confident in their judgment. This might be the case especially for participants who were determined to do well on all tests, which may explain the tendency toward an association between higher vocabulary scores and slower RTs. This hypothesis would explain why nonword decisions were made particularly slowly. There are a number of further factors that may have contributed to the group difference. Most importantly, many participants of Experiment 1 had taken part in psycholinguistic experiments before, and they were tested individually in a quiet environment. The vocational college students were novice participants and tested in a group setting, where occasional disruptions were unavoidable. In short, most likely the overall performance difference between the groups was caused by a number of linguistic and non-linguistic influences. As explained above, our goal was not to compare the two groups of students, but rather to assess whether, overall performance differences notwithstanding, similar patterns of results would be seen in both groups.

Our main interest was the relationship between individual differences in vocabulary size and lexical decision performance. For response accuracy in the lexical decision task, we found that both word frequency and vocabulary score had significant effects but did not interact. Hence, in line with findings by Yap et al. (2012) the accuracy rate increased with growing vocabulary. This effect was largely independent of word frequency. This was the case for the composite measure of vocabulary and all individual vocabulary test scores, with the exception of PPVT.

For the lexical decision latencies, we only obtained a main effect of word frequency, with faster RTs being associated with increasing word frequency but no main effect of vocabulary size.

However, the interaction between word frequency and vocabulary score was significant (see Appendix E in Supplementary Material). As expected, the word frequency effect was stronger for individuals with smaller vocabularies. This is in line with previous studies using the lexical decision task, which reported interactions between word frequency and participants' vocabulary skills (e.g., Diependaele et al., 2013). Our findings fit with the lexical entrenchment hypothesis (Diependaele et al., 2013; Brysbaert et al., 2016a) stating that the lexical representations in low-vocabulary individuals are not as robust or strong as the representations in high-vocabulary individuals due to less exposure (see also, Perfetti and Hart, 2001, 2002; Yap et al., 2009). Limited exposure is argued to have particularly strong effects on low-frequency words, resulting in smaller word frequency effects in high-vocabulary participants than in low-vocabulary participants.

## General discussion

In two experiments, we investigated the relationship between individual differences in vocabulary knowledge, assessed in a battery of vocabulary tests, and performance in a lexical decision task. One important goal of this study was to assess the merits of measuring vocabulary size comprehensively, in a battery of tests, rather than a single test; a second goal was to assess the impact of vocabulary knowledge on performance in a speeded lexical processing task. Importantly, and in contrast with most psycholinguistic studies, we tested both university students, the typical participants in psycholinguistic studies (Experiment 1), and vocational college students (Experiment 2).

Concerning the evaluation of the vocabulary test battery, the two experiments invite somewhat different conclusions. In Experiment 1, carried out with university students, the test scores were highly correlated and the composite score was similarly good in predicting performance in the lexical decision task as any of the individual tests. Both accuracy and speed were predicted by vocabulary, with increased accuracy and faster responses being associated with higher vocabulary scores. Thus, a practical recommendation from this study is that for a broad assessment of Dutch university students' vocabulary, the use of one of the standard tests, e.g., Andringa's test or the PPVT is adequate. Based on the current results no specific measure of vocabulary can be recommended as being superior over the others. It should, of course, be kept in mind that we only used a single processing task. Thus, we cannot exclude that performance in other linguistic tasks may best be predicted by a composite score based on the results of several vocabulary tests.

In Experiment 2, carried out with vocational college students, the correlations between the tests were lower than those observed in Experiment 1. The conclusions concerning the relationship between lexical decision task performance and vocabulary were a bit more varied than in Experiment 1 depending on which vocabulary test scores were used as predictors in the mixed-effects models. PPVT scores, for instance, did not predict accuracy whereas all other individual measures and the composite score of vocabulary did. When comparing the findings from Experiments 1 and 2, it has to be noted that the samples differed in size. Importantly, the more variable group in Experiment 2 is also the larger group. This supports the idea that the greater variability observed in this group (despite the larger sample size) is in fact characteristic of the population.

With regard to lexical decision speed, overall no main effect of vocabulary was found but there was a significant interaction with word frequency. However, the open antonym and synonym multiple-choice tests behaved slightly differently from the other measures of vocabulary. Participants' scores on the open antonym test did not interact with the frequency effect and the synonym multiple-choice test scores showed a significant main effect on lexical decision speed with longer RTs being associated with higher vocabulary scores (which was not significant after adjusting the significance level). The conclusions about the relationship between vocabulary and language processing might, therefore, be slightly different depending on which measure of vocabulary size is used. Thus, for testing broader samples using several vocabulary tests and combining their scores is advisable. Some of the aforementioned differences between the various vocabulary tests might of course be random fluctuations and more research especially in such more varied groups of participants is needed. Finally in our view, an important goal for further research is the development of adaptive vocabulary tests that would be suitable for all adults, regardless of their educational background.

Turning to the results of the lexical decision task, the broad pattern was the same in both experiments: There was a lexicality effect, with faster responses to words than nonwords, and a frequency effect, with faster and more accurate responses to high compared to lower frequency words. In addition, Experiment 1 yielded a main effect of vocabulary size, with participants with larger vocabularies responding faster to the stimuli than participants with smaller vocabularies. The interaction of frequency and vocabulary size that had been reported in earlier studies (Yap et al., 2012; Brysbaert et al., 2016a) was not replicated. One account for the absence of this interaction is that the sample tested in Experiment 1 was rather homogeneous with respect to their knowledge of the words used in the lexical decision task, such that main effects but not the interaction between them could be detected. The results of Experiment 2 are consistent with this proposal. For the group of vocational college students, who were expected to display a broader range of linguistic skills, an interaction of vocabulary size and frequency was obtained. Thus, it appears that this interaction can be seen when there is sufficient variability in the participants' response speed and vocabulary size. In the earlier studies, university students were tested, as in our Experiment 1 and a frequency by skills interaction was obtained. This suggests that their samples may have been more heterogeneous than ours. Alternatively, and this seems more likely, the larger frequency range in Brysbaert et al.'s (2016a) materials may be the reason for detecting the frequency × skill interaction in their data whereas we failed to elicit this interaction. Clearly further research is required to determine under which condition the vocabulary size by frequency interaction can be seen, and when it is absent. Our conclusion is that the data from Experiment 2 are largely consistent with the entrenchment hypothesis, which predicts that vocabulary size affects performance more for low- frequency than for high-frequency words. More research, using different materials, is needed to clarify why the frequency by skill interaction was not obtained in Experiment 1.

The present experiment illustrates the benefits of involving participants who are not university students in psycholinguistic experiments: The natural variability in a broader sample can be used to test specific hypotheses about, for instance, the factors determining the speed of making word/nonword decisions. In this context, it has to be noted that the variance in lexical decision speed explained by the mixed-effects models was considerably larger in Experiment 1 (*R*^2^ = 0.46) than in Experiment 2 (*R*^2^ = 0.27). Thus, more research is needed to examine potential other cognitive abilities involved in these linguistic tasks in a broad sample.

The experiments also illustrated the challenges arising in such studies. Recruiting and motivating participants can be more difficult; testing condition, for instance, in classrooms settings, can be suboptimal, and the test materials may have to be altered to accommodate novice participants. However, we strongly encourage other researchers to take up these challenges, not only in order to test specific hypotheses, but also to help the research community establish to what extent the largely university-student-based theories generalize to speakers and listeners outside of this rather small and homogeneous group.

## Ethics statement

Ethical approval for this study was obtained from the Ethics Committee of the Social Sciences Faculty of Radboud University (ECSW2014-1003-196). All subjects gave written informed consent in accordance with the Declaration of Helsinki.

## Author contributions

Substantial contributions to the conception or design of the work: NM, ZS, MB, and AM. Acquisition of data: NM. Analysis and/or interpretation of data: NM, ZS, MB, and AM. Drafting the work: NM. Revising it critically for important intellectual content: NM, ZS, MB, and AM. Final approval of the version to be published: NM, ZS, MB, and AM. Agreement to be accountable for all aspects of the work in ensuring that questions related to the accuracy or integrity of any part of the work are appropriately investigated and resolved: NM, ZS, MB, and AM.

### Conflict of interest statement

The authors declare that the research was conducted in the absence of any commercial or financial relationships that could be construed as a potential conflict of interest.

## Footnotes

^1^Five participants were 30 years or older. Inspection of their data did not suggest that they were to be treated as outliers.

^2^In addition, we ran diffusion model analyses using the fast dm algorithm (Voss and Voss, 2007; see also Ratcliff, 1978; Ratcliff et al., 2004; Gomez and Perea, 2014). Similar to reports by Yap et al. (2012) and Brysbaert et al. (2016a), many diffusion model parameters were found to be affected by vocabulary when running regression analyses in R. As the analyses did not provide a more fine-grained picture of the effect of vocabulary on word recognition performance, we do not report on them in the present paper.

^3^There are a few items with extremely high frequency values, such as *aan* (*on*; 3838.54 counts per million), *achter* (*behind*; 473.8 counts per million), and *hetzelfde* (*the same*; 193.69 counts per million). Excluding these words leads to a drop in mean word frequency from 200.16 to 22.64 counts per million, which is still higher than in some of the other tests.

^4^Participants in Experiment 1 obtained a relatively low mean score of 10.70 (*SD* = 2.86) as compared to the open antonym test (*M* = 19.29, *SD* = 2.37).
