# Supplementary material for: Vocabulary Knowledge Predicts Lexical Processing: Evidence from a Group of Participants with Diverse Educational Backgrounds
Source: Front Psychol. 2017 Jul 13;8:1164. doi: 10.3389/fpsyg.2017.01164 (PMC5507948; doi:10.3389/fpsyg.2017.01164)
Supplement: Supplementary file 1 [file DataSheet1.pdf]

Supplementary Material

Vocabulary knowledge predicts lexical processing: Evidence from a group of participants with diverse educational backgrounds

Nina Mainz*, Zeshu Shao, Marc Brysbaert & Antje S. Meyer

*** Correspondence:**
Nina Mainz
Email: nina.mainz@mpi.nl

Supplementary material: Vocabulary tests

1 Definition test

The correct responses in the table below are just examples of correct responses. Other responses might be assessed as correct too.

Trail	Target	Correct response
1	Een dier dat blaat.	schaap
2	Een dier dat knort.	varken
3	Een dier dat blaft.	hond
4	Lichaamsdeel om te zien.	oog
5	Lichaamsdeel om te proeven.	tong
6	Iemand die werkt met vlees.	slager
7	Iemand die werkt met meel.	bakker
8	Iemand die werkt met verf.	schilder
9	Iemand die werkt met eten.	kok
10	Iemand die werkt met patiënten.	dokter
11	Iemand die werkt met kip.	poelier
12	Iemand die werkt met klokken.	horlogenmaker/klokkenmaker
13	Iemand die werkt met eetgewoonten.	diëtiste
14	Vrouwtje van een kater.	poes
15	Vrouwtje van een hengst.	merrie
16	Vrouwtje van een bok.	geit
17	Vrouwtje van een reu.	teef
18	Een dier dat hinnikt.	paard
19	Lichaamsdeel om te ruiken.	neus
20	Voorwerp om mee te roeren.	lepel

2 Multiple-choice antonym test

Each target word was presented with five answer alternatives (see A, B, C, D, and E).

Trial	Target	A	B	C	D	E	Correct
1	kritiek	ordening	lof	antwoord	bejaarde	theorie	b
2	aan	terug	mild	door	uit	op	d
3	stijf	lang	vast	gezellig	flexibel	selfzuchtig	d
4	gierig	gul	vrolijk	handig	grof	koppig	a
5	bars	groot	vriendelijk	roemorig	aanwezig	intelligent	b
6	vallen	opstaan	halen	verdelen	melden	stappen	a
7	nauw	kapot	laag	wijd	tegen	donker	c
8	hetzelfde	zwaar	verschillende	blij	iemand	dergelijk	b
9	dwerg	kleinkind	kinky	reus	eender	verdrietig	c
10	aanbod	offer	toekomst	ongeluk	bieding	vraag	e
11	studiosus	flat	gesprek	luiaard	luisteraar	leraar	c
12	minst	nietig	afwezig	dezelfde	licht	meest	e
13	dapper	helder	nieuw	krachtig	bang	permantig	d
14	mals	hard	straks	soms	netjes	donzig	a
15	contra	achter	snel	echt	pro	alleen	d
16	bucolisch	klein	rustig	urbaan	duidelijk	uitvoerig	c
17	prullerig	altijd	aardig	slank	kostbaar	minuscuul	d
18	bedorven	gekookt	antiek	anzienlijk	voluit	vers	e
19	interessant	saai	prettig	zichtbaar	juist	ruim	a
20	moe	soortgelijk	samen	raak	zuur	uitgerust	e
21	pessimisme	humeur	aankomst	optimisme	mogelijkheid	plezier	c
22	aankomen	stijgen	vertrekken	vergeten	slapen	rijden	b
23	ontwaken	sluipen	inslapen	aankleden	instappen	brengen	b
24	achter	onder	hel	slap	beneden	voor	e
25	winst	verlies	bol	dorst	hoop	moed	a

3 Open antonym test

The correct responses in the table below are just examples of correct responses. Other responses might be assessed as correct too.

Trial	Target	Correct response
1	zaaien	oogsten
2	leugen	waarheid
3	nadeel	voordeel
4	minimaal	maximaal
5	unanimiteit	onenigheid
6	geforceerd	vrijwillig
7	mager	dik

8	officieus	officieel
9	lawaai	stilte
10	traag	snel
11	succes	muslukking
12	bevestiging	ontkenning
13	negatief	positief
14	absent	present
15	goedkoop	duur
16	fluisteren	schreeuwen
17	passief	actief
18	vijand	virend
19	overwinning	nederlaag
20	theoretisch	praktisch
21	ouderwets	modern
22	deficiëntie	overdaad
23	legaal	illegaal
24	tanen	toenemen
25	monochroom	bont

4 Multiple-choice synonym test

These are the five items that were added to the 20 items that the Groninger Intelligentietest is originally comprised of (Luteijn & van der Ploeg, 1983). Each target word was presented with five answer alternatives (see A, B, C, D, and E).

Trial	Target	A	B	C	D	E	Correct
1	staven	lopen	wanen	bekrachtigen	verrijken	verwaalozen	c
2	observeren	luisteren	bekijken	optellen	vermaken	trouwen	b
3	verleiding	voorstel	toeval	achtergrond	tegenvaller	bekoring	e
4	vermoeiend	eerlijk	muf	hooghartig	inspannend	dramatisch	d
5	geschikt	lastig	samen	passend	soms	duister	c

5 Open synonym test

The correct responses in the table below are just examples of correct responses. Other responses might be assessed as correct too.

Trial	Target	Correct response
1	woeden	razen
2	gekibbel	ruzie
3	verbinden	schakelen
4	overstijgen	verwinnen
5	koppig	stug

6	attitude	houding
7	latent	onderliggend
8	floreren	bloeien
9	frequent	vaak
10	gewaand	vermeend
11	permanent	altijd
12	mordicus	hardnekkig
13	morsig	smerig
14	tillen	heffen
15	obsederen	bekijken
16	beleefd	hoofs
17	vrolijk	jolig
18	emaneren	uitstralen
19	palliatief	pijnstillend
20	toezicht	controle
21	opulent	copieus
22	loyaal	trouw
23	caprice	humeur
24	wijs	slim
25	naarstig	ijverig
