# Supplementary material for: Vocabulary Knowledge Predicts Lexical Processing: Evidence from a Group of Participants with Diverse Educational Backgrounds
Source: Front Psychol. 2017 Jul 13;8:1164. doi: 10.3389/fpsyg.2017.01164 (PMC5507948; doi:10.3389/fpsyg.2017.01164)
Supplement: Supplementary file 2 [file DataSheet2.pdf]

Appendix A: Examples for all vocabulary tests.

Andringa et al.'s (2012) receptive multiple-choice test:

(1) a. Wat een vreemde *mentaliteit*! (What a strange mentality!)
    b. Een *tentatieve* lijst is te vinden op internet. (A tentative list can be found online.)

Definition test:

(2) a. Een dier dat blaft. – Hond
    (An animal that barks. – Dog)
    b. Iemand die werkt met vlees. – Slager
    (Someone who works with meat. – Butcher)

Multiple-choice antonym test:

(3) aanbod (offer)
    a. offer (victim)
    b. toekomst (future)
    c. ongeluk (accident)
    d. bieding (offer)
    e. vraag (demand)

Open antonym test:

(4) a. leugen (lie);
    Antonym: waarheid (truth)
    b. unanimiteit (unanimity);
    Antonym: verdeeldheid, onenigheid (disagreement)

Multiple-choice synonym test:

(4) vlug (quick)
    a. water (water)
    b. snel (fast)
    c. rond (round)
    d. aal (eel)
    e. haast (almost, haste)

Open synonym test:

(5) a. loyaal (loyal);
    Synonym: trouw (faithful)
    b. floreren (flourish);
    Synonym: gedijen, bloeien (thrive)

Appendix B

Reaction times for correct responses in the lexical decision task per condition for Experiments 1 and 2. For illustration purposes, frequency is shown as a categorical variable although all models are run on frequency as a continuous variable.

Group	Lexicality	Frequency	Reaction time (ms)	
			Mean	SD
Experiment 1 (university students)	Words	low	661	192
		medium	611	180
		high	591	174
		total	621	185
	Nonwords	-	718	244
Experiment 2 (vocational college students)	Words	low	855	414
		medium	781	416
		high	743	388
		total	793	409
	Nonwords	-	1035	476

Note: Low-frequency words had frequency values of less than 1 count per million in the SUBTLEX corpus (*M* = 0.36; SD = 0.27), medium-frequency items between 1 and 10 counts per million (*M* = 3.47; *SD* = 2.34), and high-frequency words between 10 and 90 counts per million (*M* = 25.24; *SD* = 20.92).

Appendix C

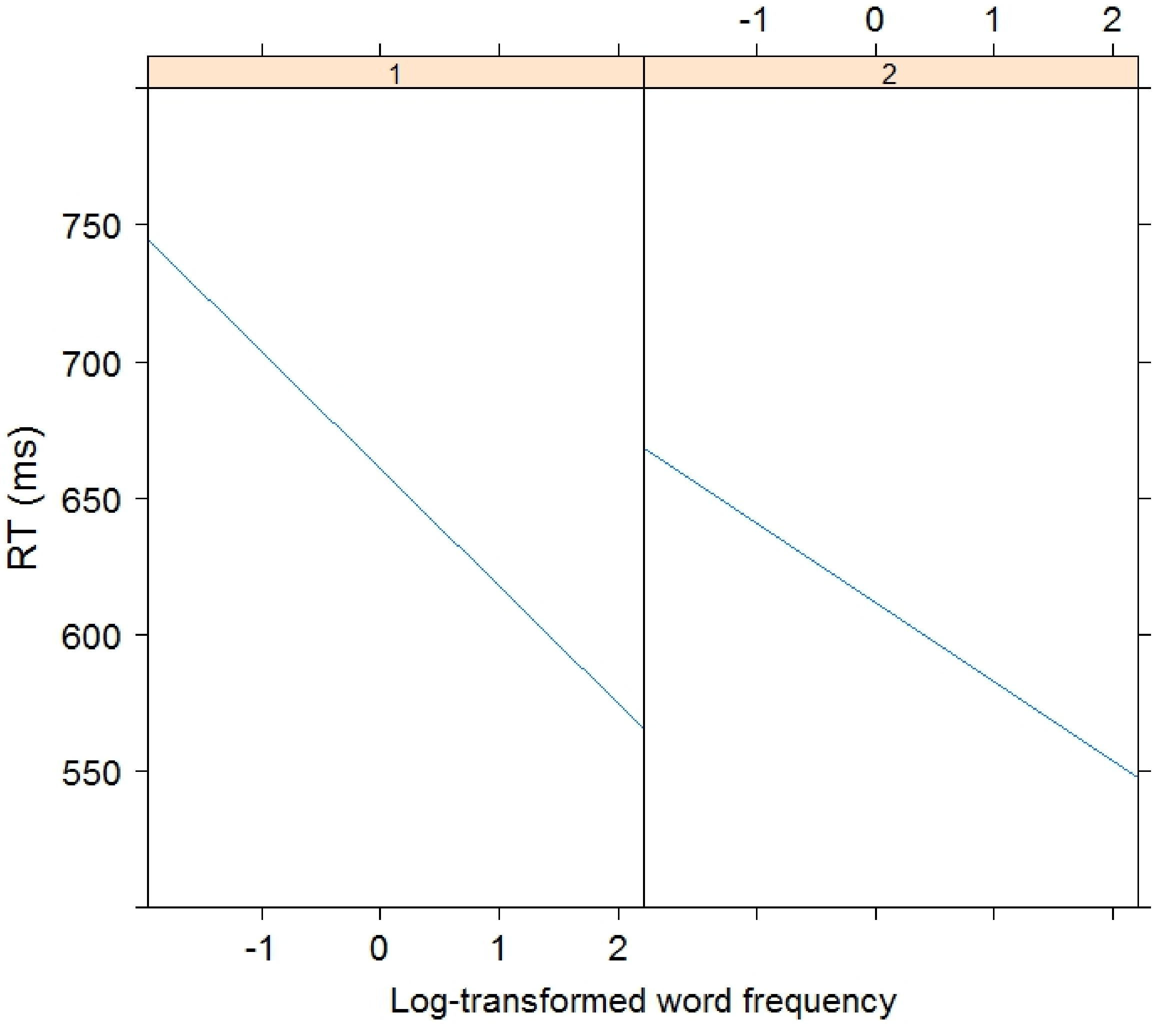

Figure 1. RT as a function of log-transformed word frequency for low- (1) vs. high-vocabulary (2) individuals. For illustration purposes, vocabulary score was transformed into a categorical variable with two levels performing a median split. The above-described model is run with vocabulary score as continuous variable.

Appendix D

P- and *t*-values for the main effects in each of the models where scores of individual vocabulary measures were used as predictor of lexical decision accuracy (Experiment 1).

Model (vocabulary test)	Variable	*t*-value	*p*-value
Definition	Vocabulary score	.91	.36
	Word frequency	7.34	<.001
	Frequency x vocabulary	.38	.70
Andringa	Vocabulary score	2.28	.02
	Word frequency	7.24	<.001
	Frequency x vocabulary	-.35	.73
Antonym MC	Vocabulary score	.02	.99
	Word frequency	7.37	<.001
	Frequency x vocabulary	1.66	.10
Antonym open	Vocabulary score	2.43	.02
	Word frequency	7.10	<.001
	Frequency x vocabulary	2.43	.70
Synonym MC	Vocabulary score	.22	.83
	Word frequency	7.36	<.001
	Frequency x vocabulary	.22	.83
Synonym open	Vocabulary score	.64	.52
	Word frequency	7.36	<.001
	Frequency x vocabulary	-.25	.80
PPVT	Vocabulary score	1.21	.23
	Word frequency	7.40	<.001
	Frequency x vocabulary	.83	.41
Composite score	Vocabulary score	1.50	.13
	Word frequency	7.33	<.001
	Frequency x vocabulary	.08	.93

Note: The Bonferroni corrected alpha level for this set of analyses is .006 (.05/8).

P- and *t*-values for the main effects in each of the models where scores of individual vocabulary measures were used as predictor of lexical decision RTs (Experiment 1).

Model (vocabulary test)	Variable	*t*-value	*p*-value
Definition	Vocabulary score	-2.75	<.01
	Word frequency	-15.26	<.001
	Frequency x vocabulary	2.0	.04
Andringa	Vocabulary score	-2.30	.02
	Word frequency	-15.30	<.001
	Frequency x vocabulary	1.5	.14
Antonym MC	Vocabulary score	-1.61	.12
	Word frequency	-15.12	<.001
	Frequency x vocabulary	.46	.64
Antonym open	Vocabulary score	-2.69	<.01
	Word frequency	-15.09	<.001
	Frequency x vocabulary	.98	.32
Synonym MC	Vocabulary score	-2.30	.02
	Word frequency	-15.14	<.001
	Frequency x vocabulary	.79	.43
Synonym open	Vocabulary score	-2.99	.004
	Word frequency	-15.18	<.001
	Frequency x vocabulary	.97	.33
PPVT	Vocabulary score	-2.05	.04
	Word frequency	-15.03	<.001
	Frequency x vocabulary	.01	.95
Composite score	Vocabulary score	-3.10	.002
	Word frequency	-15.21	<.001
	Frequency x vocabulary	1.23	.22

Note: The Bonferroni corrected alpha level for this set of analyses is .006 (.05/8).

Appendix E

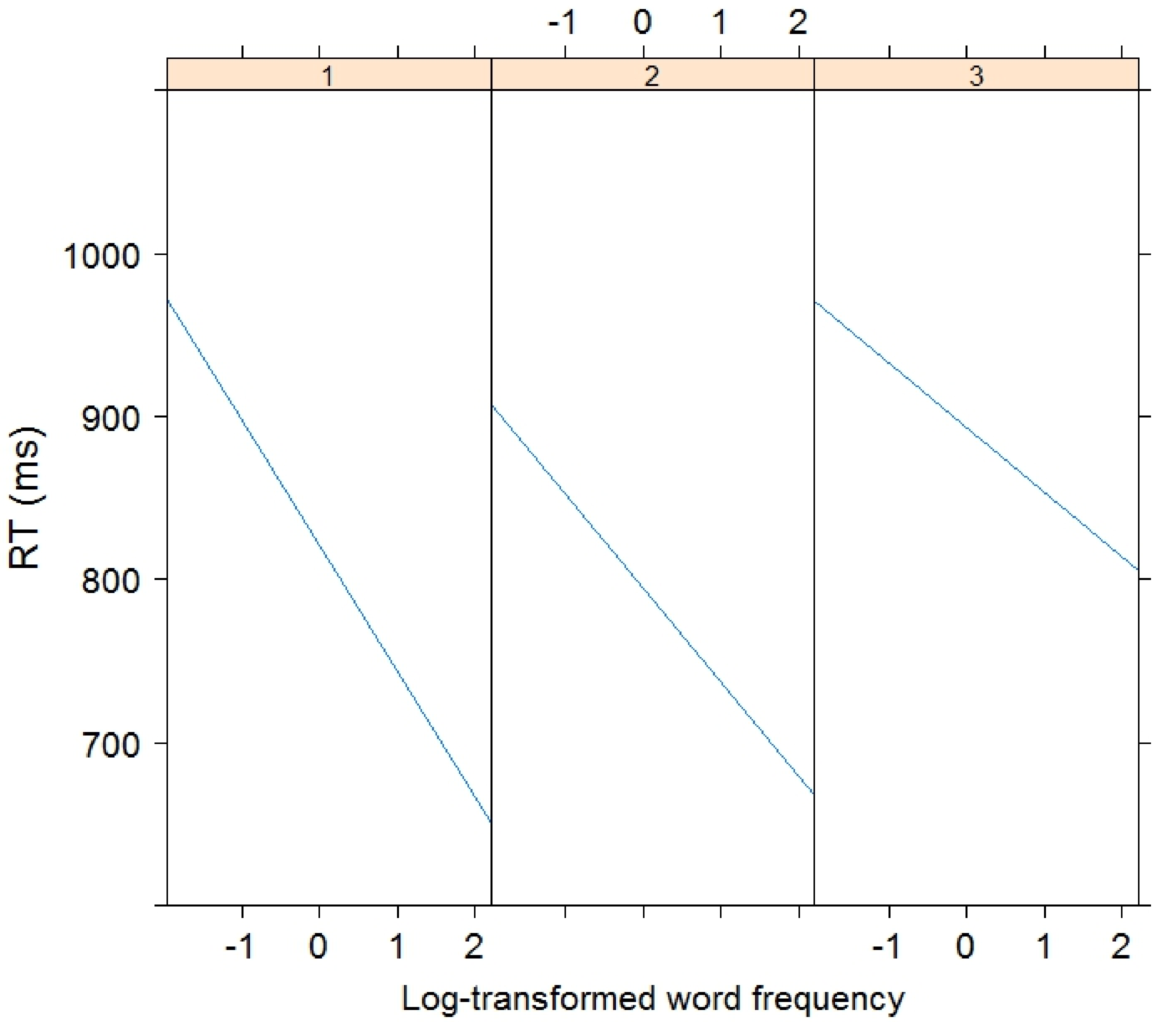

Figure 2. RT as a function of log-transformed word frequency for low- (1) vs. medium- (2) vs. high-vocabulary (3) individuals. For illustration purposes, vocabulary score was transformed into a categorical variable with three levels. The above-described model is run with vocabulary score as continuous variable.

Note: The fact that low-vocabulary individuals appear to outperform high-vocabulary individuals is probably an artefact of the different error rates. Fewer low-frequency words were included for the low- than for the high-vocabulary individuals.

Appendix F

P- and *t*-values for the main effects in each of the models where scores of individual vocabulary measures were used as predictor of lexical decision accuracy (Experiment 2). The model using participants' multiple-choice synonym scores as predictor failed to converge, even when leaving out all random slopes. That is why it is not included in the table.

Model (vocabulary test)	Variable	*t*-value	*p*-value
Definition	Vocabulary score	2.35	.01
	Word frequency	8.13	<.001
	Frequency x vocabulary	-1.49	.14
Andringa	Vocabulary score	3.81	<.001
	Word frequency	7.42	<.001
	Frequency x vocabulary	-1.64	.10
Antonym MC	Vocabulary score	4.39	<.001
	Word frequency	8.79	<.001
	Frequency x vocabulary	.79	.43
Antonym open	Vocabulary score	2.23	.03
	Word frequency	7.77	<.001
	Frequency x vocabulary	-.09	.93
PPVT	Vocabulary score	1.32	.19
	Word frequency	8.0	<.001
	Frequency x vocabulary	-.97	.33
Composite score	Vocabulary score	4.46	<.001
	Word frequency	8.72	<.001
	Frequency x vocabulary	-.89	.58

Note: The Bonferroni corrected alpha level for this set of analyses is .007 (.05/7).

P- and *t*-values for the main effects in each of the models where scores of individual vocabulary measures were used as predictor of lexical decision RTs (Experiment 2).

Model (vocabulary test)	Variable	*t*-value	*p*-value
Definition	Vocabulary score	.58	.56
	Word frequency	-13.60	<.001
	Frequency x vocabulary	3.56	<.001
Andringa	Vocabulary score	.87	.38
	Word frequency	-13.05	<.001
	Frequency x vocabulary	4.47	<.001
Antonym MC	Vocabulary score	-.28	.78
	Word frequency	-13.90	<.001
	Frequency x vocabulary	2.15	.03
Antonym open	Vocabulary score	-1.39	.16
	Word frequency	-12.32	<.001
	Frequency x vocabulary	1.27	.20
Synonym MC	Vocabulary score	2.46	.01
	Word frequency	-12.53	<.001
	Frequency x vocabulary	2.61	.009
PPVT	Vocabulary score	1.40	.16
	Word frequency	-12.52	<.001
	Frequency x vocabulary	3.06	.002
Composite score	Vocabulary score	.90	.37
	Word frequency	-14.99	<.001
	Frequency x vocabulary	4.10	<.001

Note: The Bonferroni corrected alpha level for this set of analyses is .007 (.05/7).
